# Work–family conflict and self-rated health among Japanese workers: How household income modifies associations

**DOI:** 10.1371/journal.pone.0169903

**Published:** 2017-02-16

**Authors:** Tomoko Kobayashi, Kaori Honjo, Ehab Salah Eshak, Hiroyasu Iso, Norie Sawada, Shoichiro Tsugane, for the Japan Public Health Center-based Prospective Study for the Next Generation (JPHC-NEXT) Study Group

**Affiliations:** 1 Public Health, Department of Social Medicine, Graduate School of Medicine, Osaka University, Osaka, Japan; 2 Department of Public Health and Preventive Medicine, Minia University, Minia, Egypt; 3 Epidemiology and Prevention Group, Center for Public Health Sciences, National Cancer Center, Tokyo, Japan; Hamamatsu Ika Daigaku, JAPAN

## Abstract

To examine associations between work–family conflict and self-rated health among Japanese workers and to determine whether the associations differed by household income. Data was derived from the Japan Public Health Center-based Prospective Study for the Next Generation in Saku area in 2011–2012 (7,663 men and 7,070 women). Multivariate odds ratios (ORs) and 95% confidence intervals (CIs) for poor self-rated health by work–family conflict consisting of two dimensions (work-to-family and family-to-work conflicts) were calculated by gender and household income. Multivariate ORs of high work-to-family and family-to-work conflicts for poor self-rated health were 2.46 (95% CI; 2.04–2.97) for men and 3.54 (95% CI; 2.92–4.30) for women, with reference to the low work-to-family and family-to-work conflicts (*p*-value for gender interaction = 0.02). Subgroup analysis indicated that health effects of work–family conflict were likely to be more evident in the low income group only among women. Work–family conflict was associated with poor self-rated health among middle-aged Japanese men and women; its health impact was relatively stronger among women, and particularly economically disadvantaged women.

## Introduction

The work–family literature on “work–family conflict” has expanded steadily over the past few decades, along with an increase in dual-earner couples, single-parent families, and caregiving needs of elderly people.[1–4] Work–family conflict is generally defined as “a form of inter-role conflict in which the role pressures from the work and family domains are mutually incompatible in some respect.”[5] Importantly, there is growing evidence linking work–family conflict and various health outcomes such as psychiatric disorders,[6] mental disorders,[7] depression,[8, 9] psychological distress,[10] mental health,[11] health behaviors (e.g., smoking, unhealthy eating habits, and heavy alcohol consumption),[12] drinking behaviors,[8, 13] physical and mental functioning,[14] smoking,[15] sleep problems,[16] sleep medication,[17] and poor self-rated health.[18–21]

However, it is still unclear if there is a gender difference in the health effects of work–family conflict. In most societies, the household responsibilities of women persist despite more women entering and playing a greater role in the workforce.[22] The demands of household duties and responsibilities often increase work–family conflicts for women.[3] In line with this, a Swedish representative longitudinal study reported that women experienced work–family conflict slightly more often than men, and an increased risk for poor self-rated health existed only among women.[19] On the other hand, a cross-sectional study on self-rated health in the United Kingdom showed that work–family conflict associated with reporting poor self-rated health was equally observed among men and women.[18] Another cross-sectional study with municipal employees in Finland also identified that work-to-family and family-to-work conflicts were associated with poor self-rated health among both men and women.[20]

One of the salient features of Japanese society is the male breadwinner model: men work outside the home to provide an income for their family, while women stay at home as housewife, providing care for children and the elderly. This model persists to this day in Japanese society.[23] For example, the time spent by Japanese men on housework (less than 1 hour per week) has not changed substantially over the past two decades[24] despite an increase in women’s social participation in the workforce. Arguably then, Japanese working women may experience more conflicts than men, as the demands of their work role conflict with the demands of their family role.

Furthermore, a recent cross-sectional study among Brazilian civil servants have shown that the association between work–family conflict and self-rated health differed by educational level only among women.[21] While few studies have focused on how socioeconomic conditions affect work–family conflict and self-rated health,[21] the association between work–family conflict and self-rated health may differ by socioeconomic conditions. Thus, the impacts of work–family conflict on self-rated health would also differ by household income.

There is some evidence available on work–family conflict in Japan;[9–12, 14, 16] in these studies, Japanese workers with high work–family conflict reported poorer self-rated health. However, most of this evidence was derived from limited samples such as civil servants of local government,[11, 12, 14, 16] regularly employed information technology engineers,[9] and dual-earner couples in a metropolitan area.[10] No studies have been conducted for self-rated health with general community residents under various working conditions.

The objective of this study was to investigate the associations of work–family conflict and self-rated health among community residents in Japan. We aimed to answer the following specific research questions:

1. Are there any associations between work–family conflict and self-rated health among Japanese working men and women?
2. Are the associations noted above modified by socioeconomic conditions (i.e., household income level)?

## Methods

### Ethics statement

All participants provided written informed consent. This study was approved by the institutional review board of the National Cancer Center Japan and Osaka University.

### Participants

The Japan Public Health Center-based prospective Study for the Next Generation (JPHC-NEXT Study) was initiated in 2011, and a baseline survey is ongoing until December 31, 2016. Data in this study were derived from the JPHC-NEXT in the Saku area (JPHC-NEXT Saku). We established a population-based cohort of 55,571 residents aged 40–74 who registered their address in the Saku area (Saku city, Sakuho, Koumi towns and Minami-aiki, Kita-aiki, Mminami-maki, Kawakami villages) supervised by the Saku Public Health Center (PHC) areas in 2011–2012. A self-administered questionnaire was submitted to all cohort participants, who were asked to report their lifestyles, personal medical histories, and socio-demographic situations. The questionnaire was distributed mostly by hand, but partly by mail, in 2011–2012. Incomplete answers were supplemented by telephone interview. From a total of 55,571 residents, 31,395 agreed to participate in the JPHC-NEXT Saku Study (response rate = 57%).

We excluded people over 65 years old (*n* = 9,149) and people who were unemployed (*n* = 8,507) to restrict the participants to workers, and excluded people who had medical histories of cancer (*n* = 774), cardiovascular disease (*n* = 2,784), stroke (*n* = 678), or depression (*n* = 569) to minimize the possible effects of initial health status. Of the remaining 17,093 people, we further excluded the following: those who did not supply information on self-rated health (*n* = 142), or any items concerning work–family conflict (*n* = 1,267); those who reported “not applicable” regarding any items of work–family conflict (*n* = 997). The final number of participants included in the analysis was 14,733 (7,663 men and 7,070 women).

### Measures

#### Work–family conflict

We assessed work–family conflict with the following eight items adapted from the National Study of Midlife Development in the United States:[1, 11] (i) “Your job reduces the amount of time you can spend with the family”; (ii) “Problems at work make you irritable at home”; (iii) “Your work involves a lot of travel away from home”; (iv) “Your job takes so much energy you do not feel up to doing things that need attention at home”; (v) “Family matters reduce the time you can devote to your job”; (vi) “Family worries about problems distract you from your work”; (vii) “Family activities stop you getting the amount of sleep you need to do your job well”; (viii) “Family obligations reduce the time you need to relax or be yourself.” Of these items, the first four identified work-to-family conflict (WF conflict), while the last four identified family-to-work conflict (FW conflict). Respondents chose one of three categories (0 = never, 1 = to some extent, 2 = often) for each question, and a total score, ranging from 0 to 8, was calculated by summing all of the items. We divided each of the total scores into two categories based on the median (WF conflict: 0–2 / 3–8; FW conflict: 0 / 1–8) [20] and then created four categories: 1) low WF conflict and low FW conflict; 2) high WF conflict and low FW conflict; 3) low WF conflict and high FW conflict; and, 4) high WF conflict and high FW conflict. The internal consistency of work–family conflict was deemed acceptable (Cronbach’s alpha = 0.77 for men and 0.79 for women).

#### Self-rated health

We measured self-rated health as an outcome using the single questionnaire item: “How would you describe your overall state of health?” We categorized responses as follows to indicate a level of self-rated health: 0 = excellent, very good, or good (better health); 1 = not very good, or not good (poor health).[25, 26]

#### Covariates

We considered the following variables as relevant confounders in our analyses: age (continuous); household equivalent income (low, intermediate, and high); educational attainment (junior high/high school, some college, and college or more); employment status (regular worker, non-regular worker including contract workers, temporary workers, and part-time workers, and self-employed); occupation (white-collar job: professional, management, office work, sales, and service; blue-collar job: security, farming/forestry/fishery, transportation, and labor service);[27] domestic role (non-married/married without children under 15 years old, non-married/married with children under 15 years old);[11, 14] medical histories of hypertension, diabetes mellitus, and hypercholesterolemia identified from responses (yes or no) to the questionnaire; and social support (low and high).[28] Social support was measured by the 7-item self-report ENRICHD Social Support Instrument.[28] Responses of social support regarding items 1 to 6 were rated on a 5-point scale (0 = none of the time, 4 = all of the time) and the response to item 7 was dichotomized (0 = Yes, 1 = No). The total score, which ranged from 0 to 25, was dichotomized based on the median. Income data were obtained using the following six categories of annual household income (in million yen) before taxes: < 3, 3–5.99, 6–8.99 9–11.99, 12–14.99, ≤15. Adopting the median value of each category, we calculated household equivalent income by dividing household income by the square root of total household members.[29] Finally, we categorized household equivalent income into three categories (low, intermediate, and high) based on tertile distributions.

### Statistical analysis

Proportions of work–family conflict, poor self-rated health, and other variables were reported. We compared the proportion of those variables by self-rated health using a chi-square test. We performed multivariate logistic regression analysis to examine the associations between WF conflict, FW conflict, and work–family conflict and poor self-rated health according to gender. We adjusted for sociodemographic factors such as age, household equivalent income, educational attainment, employment status, and occupation (Model 2) and further adjusted hypothesized confounding factors such as domestic role, social support, and medical histories of hypertension, diabetes mellitus, and hypercholesterolemia (Model 3). Additionally, we conducted subgroup analysis by household equivalent income group. We tested statistical interaction by using cross-product terms for work–family conflict and gender or household equivalent income. All analyses were performed using Stata version 12.1 (Stata Corp LP, College Station, TX, USA).

## Results

Table 1 shows sex-specific proportions of work–family conflict and demographic characteristics, stratified by self-rated health. A total of 1,065 (13.9%) men and 1,249 (17.7%) women rated their health as poor: gender difference in poor self-rated health (*p* <0.001). The proportion of high WF conflict was 36.6% for men and 28.4% for women, while those of high FW conflict were 36.4% for men and 56.8% for women. The proportions of men and women with high WF conflict and high FW conflict were 19.8% and 22.0%, respectively. High work–family conflict was more prevalent among those with poor self-rated health than those with good self-rated health. Of the 8 items of work–family conflict, the proportion of men who responded “often” to the question (iii), “Your work involves a lot of travel away from home,” was higher than that of women in the highest work–family conflict group. By contrast, the proportion of women who responded “often” to the question (vii), “Family activities stop you getting the amount of sleep you need to do your job well,” and (viii) “Family obligations reduce the time you need to relax or be yourself,” were higher than those of men (see S1 Table). Furthermore, men reporting poor self-rated health were more likely to be younger and had more domestic roles (married with children under 15 years old), whereas women reporting poor self-rated health were more likely to be less educated, to be regular workers, and to have fewer domestic roles. Both men and women who reported having lower social support and medical histories of hypertension, diabetes mellitus, and hypercholesterolemia were more likely to report poor self-rated health.

**Table 1:** Sex specific work-family conflict and demographic characteristics according to self-rated health.

	Men							Women						
	All		Good health		Poor health			All		Good health		Poor health		
	*n* = 7663		*n* = 6598	(86.1%)	*n* = 1065	(13.9%)		*n* = 7070		*n* = 5821	(82.3%)	*n* = 1249	(17.7%)	
Characteristics	N	%	N	%	N	%	p value*	N	%	N	%	N	%	p value*
Work-to-family conflict (WF conflict)							< .001							< .001
Low	4858	63.4	4334	65.7	524	49.2		5062	71.6	4368	75.0	694	55.6	
High	2805	36.6	2264	34.3	541	50.8		2008	28.4	1453	25.0	555	44.4	
Family-to-work conflict (FW conflict)							< .001							< .001
Low	4873	63.6	4331	65.6	542	50.9		3056	43.2	2681	46.1	375	30.0	
High	2790	36.4	2267	34.4	523	49.1		4014	56.8	3140	53.9	874	70.0	
Work–family conflict							< .001							< .001
Low WF and low FW conflicts	3585	46.8	3255	49.3	330	31.0		2602	36.8	2331	40.0	271	21.7	
Low WF and high FW conflicts	1273	16.6	1079	16.4	194	18.2		2460	34.8	2037	35.0	423	33.9	
High WF and low FW conflicts	1288	16.8	1076	16.3	212	19.9		454	6.4	350	6.0	104	8.3	
High WF and high FW conflicts	1517	19.8	1188	18.0	329	30.9		1554	22.0	1103	19.0	451	36.1	
Age							0.01							< .01
40–49	2926	38.2	2485	84.9	441	15.1		2849	40.3	2377	40.8	472	37.8	
50–59	3158	41.2	2720	86.1	438	13.9		3010	42.6	2424	41.6	586	46.9	
60–64	1579	20.6	1393	88.2	186	11.8		1211	17.1	1020	17.5	191	15.3	
Household equivalent income							0.11							0.36
Low	2214	28.9	1883	28.5	331	31.1		2791	39.5	2280	39.2	511	40.9	
Intermediate	2843	37.1	2454	37.2	389	36.5		2016	28.5	1658	28.5	358	28.7	
High	2085	27.2	1819	27.6	266	25.0		1766	25.0	1472	25.3	294	23.5	
Missing	521	6.8	442	6.7	79	7.4		497	7.0	294	7.1	86	6.9	
Educational attainment							0.12							0.01
Junior high/high school	3951	51.6	3389	51.4	562	52.8		3512	49.7	2879	49.5	633	50.7	
Some college	1608	21.0	1371	20.8	237	22.3		2929	41.4	2401	41.3	528	42.3	
College or more	2026	26.4	1771	26.8	255	23.9		559	7.9	488	8.4	71	5.7	
Missing	78	1.0	67	1.0	11	1.0		70	1.0	53	0.9	17	1.4	
Employment status							0.24							< .001
Regular worker	4665	60.9	3993	60.5	672	63.1		2371	33.5	1888	32.4	483	38.7	
Non-regular worker	914	11.9	799	12.1	115	10.8		3517	49.8	2930	50.3	587	47.0	
Self-employed	2084	27.2	1806	27.4	278	26.1		1182	16.7	1003	17.2	179	14.3	
Occupation							0.54							0.38
White-collar job	5194	67.8	4490	68.1	704	66.1		5090	72	4177	71.8	913	73.1	
Blue-collar job	2106	27.5	1809	27.4	297	27.9		1325	18.7	913	18.9	224	17.9	
Missing	363	4.7	299	4.5	64	6.0		655	9.3	224	9.3	112	9.0	
Domestic role							< .001							< .01
Non-married without children aged under 15	1176	15.4	984	14.9	192	19.0		957	13.5	755	13.0	202	16.2	
Married without children aged under 15	4081	53.3	3581	54.3	500	47.0		4085	57.8	3345	57.5	740	59.2	
Non-married with children aged under 15	60	0.8	52	0.8	8	0.8		210	3.0	177	3.0	33	2.6	
Married with children aged under 15	2309	30.1	1948	29.5	361	33.9		1793	25.4	1524	26.2	269	21.5	
Missing	37	0.5	33	89.2	4	0.4		25	0.4	20	0.3	5	0.4	
Social support							< .001							< .001
Low	4276	55.8	3565	54.0	711	66.8		3454	48.9	2722	46.8	732	58.6	
High	3387	44.2	3033	46.0	354	33.2		3616	51.2	3099	53.2	517	41.4	
Hypertension							< .01							< .001
No	6635	86.6	5743	87.0	892	83.8		6383	90.3	5322	91.4	1061	85.0	
Yes	1028	13.4	855	13.0	173	16.2		687	9.7	499	8.6	188	15.1	
Diabetes mellitus							< .001							< .001
No	7219	94.2	6245	94.7	974	91.5		6920	97.9	5722	98.3	1198	95.9	
Yes	444	5.8	353	5.4	91	8.5		150	2.1	1198	1.7	51	4.1	
Hypercholesterolemia							< .01							< .001
No	6779	88.5	5868	88.9	911	85.5		6367	90.1	5291	90.9	1076	86.2	
Yes	884	11.5	730	11.1	154	14.5		703	9.9	530	9.1	173	13.9	

WF, work-to-family; FW, family-to-work.

*p value for Chi-squared test.

Table 2 shows gender-specific adjusted odds ratios (ORs) for poor self-rated health associated with work–family conflict. We identified statistically significant associations between work–family conflict and poor self-rated health among both men and women. High WF conflict, as well as high FW conflict, was associated with poor self-rated health among both men and women. The adjusted ORs (95% confidence interval [CI]) for poor self-rated health in the high WF conflict and high FW conflict group were 2.46 (95% CI, 2.04–2.97) for men and 3.54 (95% CI 2.92–4.30) for women, with reference to the low WF conflict and low FW conflict group. The association between work–family conflict and poor self-rated health among women was relatively stronger than that among men (interaction for *p* = 0.02).

**Table 2:** Odds ratios (OR) for poor self-rated health associated with work–family conflict, separately by gender.

	Model 1		Model 2		Model 3	
	OR	(95% CI)	OR	(95% CI)	OR	(95% CI)
**Men**						
**Work-to-family conflict**						
Low	1.00		1.00		1.00	
High	1.98	(1.73–2.25)	1.98	(1.71–2.29)	1.84	(1.59–2.14)
**Family-to-work conflict**						
Low	1.00		1.00		1.00	
High	1.84	(1.62–2.10)	1.83	(1.59–2.10)	1.68	(1.45–1.94)
**Work Family conflict group**						
Low WF and low FW conflicts	1.00		1.00		1.00	
Low WF and high FW conflicts	1.77	(1.47–2.15)	1.76	(1.43–2.16)	1.63	(1.32–2.01)
High WF and low FW conflicts	1.94	(1.61–2.34)	1.93	(1.57–2.37)	1.82	(1.48–2.24)
High WF and high FW conflicts	2.73	(2.31–3.23)	2.74	(2.28–3.29)	2.46	(2.04–2.97)
**Women**						
**Work to Family conflict**						
Low	1.00		1.00		1.00	
High	2.40	(2.12–2.73)	2.48	(2.15–2.86)	2.37	(2.05–2.74)
**Family to Work conflict**						
Low	1.00		1.00		1.00	
High	1.99	(1.74–2.27)	2.04	(1.76–2.36)	1.99	(1.71–2.31)
**Work Family conflict group**						
Low WF and low FW conflicts	1.00		1.00		1.00	
Low WF and high FW conflicts	1.79	(1.52–2.10)	1.81	(1.50–2.17)	1.77	(1.47–2.14)
High WF and low FW conflicts	2.56	(1.99–3.29)	2.52	(1.90–3.34)	2.39	(1.79–3.18)
High WF and high FW conflicts	3.52	(2.98–4.16)	3.70	(3.07–4.67)	3.54	(2.92–4.30)

CI, confidence interval; OR, odds ratio; WF, work-to-family; FW, family-to-work.

Model 1: crude model.

Model 2: adjusted for age, household equivalent income, educational attainment, employment status, and occupation.

Model 3: Model 2 + domestic role, social support, and medical histories of hypertension, diabetes mellitus, and hypercholesterolemia.

The subgroup analysis by household income showed that the association between work–family conflict and self-rated health was modified by household equivalent income only among women in Table 3 (interaction for *p* = 0.01). The adjusted ORs (95% CI) for poor self-rated health of the women who reported high WF conflict and high FW conflict with reference to those reporting low WF conflict and low FW conflict, were 4.63 (95% CI, 3.42–6.27) in the lowest household income group, 3.49 (95% CI, (2.45–4.99) in the intermediate household income group, and 2.45 (95% CI, 1.72–3.50) in the highest household income group. For men, no modification by household income was observed.

**Table 3:** Odds ratios (OR) for poor self-rated health associated with work–family conflict, by household equivalent income.

	Household equivalent income									p value for interaction
	Low			Intermediate			High			
	Poor health/N	OR^a^	(95% CI)	Poor health/N	OR^a^	(95% CI)	Poor health/N	OR^a^	(95% CI)	
**Men**										
**Work Family conflict group**										
Low WF and low FW conflicts	100/1064	1.00		108/1241	1.00		89/975	1.00		0.92
Low WF and high FW conflicts	87/454	2.12	(1.52–2.98)	62/450	1.51	(1.06–2.15)	33/299	1.21	(0.79–1.87)	
High WF and low FW conflicts	47/281	1.90	(1.27–2.83)	87/510	2.12	(1.53–2.93)	59/415	1.45	(1.00–2.10)	
High WF and high FW conflicts	97/415	2.57	(1.83–3.61)	132/642	2.46	(1.82–3.31)	85/396	2.43	(1.72–3.44)	
**Women**										
**Work Family conflict group**										
Low WF and low FW conflicts	95/1026	1.00		65/644	1.00		81/681	1.00		0.01
Low WF and high FW conflicts	190/1028	2.07	(1.55–2.77)	130/769	1.84	(1.30–2.61)	76/516	1.36	(0.95–1.96)	
High WF and low FW conflicts	42/155	3.67	(2.36–5.71)	24/121	1.96	(1.12–3.44)	26/144	1.59	(0.95–2.67)	
High WF and high FW conflicts	184/582	4.63	(3.42–6.27)	139/482	3.49	(2.45–4.99)	111/425	2.45	(1.72–3.50)	

CI, confidence interval; OR, odds ratio; WF, work-to-family; FW, family-to-work.

^a^Adjusted for age, educational attainment, employment status, occupation, domestic role, social support, and medical histories of hypertension, diabetes mellitus, and hypercholesterolemia.

## Discussion

The present study shows that higher WF conflict and higher FW conflict are associated with increased odds of having poor self-rated health among both men and women. The work–family conflict represented by a combination of WF conflict and FW conflict also showed a statistically significant association with self-rated health, which was more evident among women than men. Subgroup analysis by household income indicated that the associations between work–family conflict and self-rated health were modified only among women; a stronger association between work–family conflict and self-rated health was observed in the lower household income group among women, while no significant differences were identified among men.

Our results are in line with previous research showing harmful effects of higher work–family conflict on poor self-rated health.[18–21] There have been a limited number of studies on the health effects of work–family conflict among Japanese populations. One cross-sectional study indicated that higher work–family conflicts were associated with an increased probability of having poor self-rated mental health among both men and women.[11] Another study also showed that higher work–family conflict was associated with poorer psychological health.[9,10] It is suggested that increased inter-role conflict in which the role pressures from work and family domains can reduce time for sleep or leisure activities, and thus increase psychological stress, consequently affect physical health conditions.[3]

As we noted previously, the evidence for a gender difference in the effects of work–family conflict on self-rated health remains mixed.[18–21] In this study, we observed a significant gender difference in the effect of work–family conflict on self-rated health: the relatively stronger association between work–family conflict and self-rated health among women. While we did not examine the mechanisms underlying this gender difference, we did explore gender differences in the nature of the work–family conflict and found substantive gender-specific responses to the questions related to work–family conflict in our data; the proportion of men who responded “often” to the question about work-to-family conflict due to a lot of business travel was higher than that of women. By contrast, the proportion of women who responded “often” to the question about family-to-work conflict due to family activities and family obligations were higher than those of men (see S1 Table). Given the materially imbalanced household duties of men and women, women will arguably find it more challenging to juggle work- and family-related demands.[22] Thus, the dissimilarity in the nature of work–family conflict underlying normalized gendered roles in Japan may help explain the gender-based differences in the health effects of work–family conflict.

Contrary to our hypothesis, we found similar proportions of men and women who reported having high work–family conflict. However, we found that men had higher WF conflict, while women had higher FW conflict. Strong gendered social roles such as the male breadwinner model may affect these differences. Women tend to give priority to homemaker and maternal roles and thus may be more likely to feel FW conflict, while men tend to emphasize responsibilities in their breadwinner role and thus are more likely to experience WF conflict.

Furthermore, we showed higher effects of work–family conflict on self-rated health in the low income group, compared with those in the intermediate or high income group among women. One possible explanation is that women with low household incomes might bear much of the responsibility for their family due to the economic constrains. For example, the nationally representative surveys of Japan have shown that an unhealthy nutrient intake was associated with lower household expenditure.[30] In addition, despite a universal health care insurance system in Japan, people with low incomes are likely to have poorer access to outpatient care and more serious health conditions than people with high incomes.[31] Given Japan’s gendered social roles, there might be some possibility that Japanese women with low incomes prioritize needs of their families such as food and health issues. As a result, women perceived high work–family conflict in the low income group seem to have reported poorer health status. Further studies are needed to clarify how household income modifies the associations between work–family conflicts and self-rated health by taking into account food, medical access, and other residual confounders.

To our knowledge, this is the first study to examine the association between work–family conflict and self-rated health in the general Japanese population. However, several limitations should also be noted. First, we cannot exclude the possibility of reverse causation because of the cross-sectional design. To reduce this possibility, we excluded people who had medical histories of cancer, cardiovascular disease, stroke, and depression to avoid the possible effects of initial health status. Supplementary analysis conducted after further excluding people who had medical histories of hypertension, diabetes mellitus, and hypercholesterolemia did not change our conclusion. In addition, supplementary analysis limited items of work–family conflicts (see S2 Table) showed similar results to those in Table 2, although we obtained somewhat attenuated results among women. Second, we did not have information on work-related factors (e.g., working hours and job demands) that are considered relevant to work-to-family conflict,[3] and on the content or time spent on child caring and household work. It is likely that these work- and family-related factors vary by gender and income, and thus future studies are needed that take such work-related factors into account. Third, as both exposure and outcome variables were assessed by self-reported questionnaires, spurious correlations could have occurred if respondents answered questions consistently but not accurately. Although self-rated health has proved to be an independent predictor of mortality, over and above other health status indicators and other relevant covariates known to predict mortality,[25, 26] future prospective studies are needed to test the robustness of our study by using not subjective outcomes such as mobility or mortality. Furthermore, the possibility of missing bias cannot be ruled out due to self-reported assessment of both exposure and outcome. Excluded participants were likely to be older, have less household income compared to our study population but no significant difference in self-rated health. In our study, missing of work–family conflicts seemed to be non-differential to self-rated health, which may have attenuated the present results. Finally, we must generalize our results cautiously to other populations, because our sample was restricted to residents of one public center area in Japan and the response rate was not high (57%).

## Conclusions

In this study, we showed that higher work–family conflict was associated with poor self-rated health among both men and women in the general Japanese population. These associations were stronger among women than among men, and in particular, among economically disadvantaged women. The present findings may help advance understanding of gender differences in the associations between work–family conflict and poor self-rated health.

## Supporting information

S1 Table Distribution of work-family conflict among men and women. (DOCX)

S2 Table Odds ratios (OR) for poor self-rated health associated with work–family conflict using limited items, separately by gender. (DOCX)
