# Supplementary material for: Work–family conflict and self-rated health among Japanese workers: How household income modifies associations
Source: PLoS One. 2017 Feb 16;12(2):e0169903. doi: 10.1371/journal.pone.0169903 (PMC5312934; doi:10.1371/journal.pone.0169903)
Supplement: S1 Table — (DOCX) [file pone.0169903.s001.docx]

**S1 Table.** Distribution of work-family conflict among men and women

|  | MEN | | | | | | | |  | WOMEN | | | | | | | |
| --- | --- | --- | --- | --- | --- | --- | --- | --- | --- | --- | --- | --- | --- | --- | --- | --- | --- |
|  | Low WF and low FW conflicts (*n* =3585) | | Low WF and high FW conflicts (*n* = 1273) | | High WF and low FW conflicts (*n* =1288) | | High WF and high FW conflicts (*n* =1517) | |  | Low WF and low FW conflicts (*n* =2602) | | Low WF and high FW conflicts (*n* = 24603) | | High WF and low FW conflicts (*n* =454) | | High WF and high FW conflicts (*n* =1554) | |
| **Work-to-family** | N | % | N | % | N | % | N | % |  | N | % | N | % | N | % | N | % |
| Item 1 |  |  |  |  |  |  |  |  |  |  |  |  |  |  |  |  |  |
| Never | 2,581 | 72.0 | 639 | 50.2 | 60 | 4.7 | 44 | 2.9 |  | 2,118 | 81.4 | 1458 | 59.3 | 49 | 10.8 | 76 | 4.9 |
| Some extent | 877 | 24.5 | 586 | 46.0 | 578 | 44.9 | 753 | 49.6 |  | 445 | 17.1 | 955 | 38.8 | 230 | 50.7 | 830 | 53.4 |
| Often | 127 | 3.5 | 48 | 3.8 | 650 | 50.5 | 720 | 47.5 |  | 39 | 1.5 | 47 | 1.9 | 175 | 38.6 | 648 | 41.7 |
| Item 2 |  |  |  |  |  |  |  |  |  |  |  |  |  |  |  |  |  |
| Never | 2,952 | 82.3 | 775 | 60.9 | 298 | 23.1 | 164 | 10.8 |  | 2,027 | 77.9 | 1511 | 61.4 | 38 | 8.4 | 98 | 6.3 |
| Some extent | 606 | 16.9 | 480 | 37.7 | 804 | 62.4 | 1,036 | 68.3 |  | 541 | 20.8 | 916 | 37.2 | 295 | 65.0 | 1,057 | 68.0 |
| Often | 27 | 0.8 | 18 | 1.4 | 186 | 14.4 | 317 | 20.9 |  | 34 | 1.3 | 33 | 1.3 | 121 | 26.7 | 399 | 25.7 |
| Item 3 |  |  |  |  |  |  |  |  |  |  |  |  |  |  |  |  |  |
| Never | 3,269 | 91.2 | 1,145 | 90.0 | 531 | 41.2 | 711 | 46.9 |  | 2,555 | 98.2 | 2,416 | 98.2 | 314 | 69.2 | 1138 | 73.2 |
| Some extent | 294 | 8.2 | 123 | 9.7 | 508 | 39.4 | 626 | 41.3 |  | 43 | 1.7 | 43 | 1.8 | 124 | 27.3 | 378 | 24.3 |
| Often | 22 | 0.6 | 5 | 0.4 | 249 | 19.3 | 180 | 11.9 |  | 4 | 0.2 | 1 | 0.0 | 16 | 3.5 | 38 | 2.5 |
| Item 4 |  |  |  |  |  |  |  |  |  |  |  |  |  |  |  |  |  |
| Never | 3,357 | 93.6 | 1,074 | 84.4 | 443 | 34.4 | 313 | 20.6 |  | 2,394 | 92.0 | 2,007 | 81.6 | 94 | 20.7 | 200 | 12.9 |
| Some extent | 222 | 6.2 | 195 | 15.3 | 662 | 51.4 | 979 | 64.5 |  | 204 | 7.8 | 443 | 18.0 | 276 | 60.8 | 1043 | 67.1 |
| Often | 6 | 0.2 | 4 | 0.3 | 183 | 14.2 | 225 | 14.8 |  | 4 | 0.2 | 10 | 0.4 | 84 | 18.5 | 311 | 20.0 |
| **Family-to-work** |  |  |  |  |  |  |  |  |  |  |  |  |  |  |  |  |  |
| Item 5 |  |  |  |  |  |  |  |  |  |  |  |  |  |  |  |  |  |
| Never | 3,585 | 100.0 | 699 | 54.9 | 1,288 | 100.0 | 653 | 43.1 |  | 2,602 | 100.0 | 1414 | 57.5 | 454 | 100.0 | 671 | 43.2 |
| Some extent | – | – | 518 | 40.7 | – | – | 773 | 51.0 |  | – | – | 928 | 37.7 | – | – | 754 | 48.5 |
| Often | – | – | 56 | 4.4 | – | – | 91 | 6.0 |  | – | – | 118 | 4.8 | – | – | 129 | 8.3 |
| Item 6 |  |  |  |  |  |  |  |  |  |  |  |  |  |  |  |  |  |
| Never | 3,585 | 100.0 | 439 | 34.5 | 1,288 | 100.0 | 355 | 23.4 |  | 2,602 | 100.0 | 1119 | 45.5 | 454 | 100.0 | 418 | 26.9 |
| Some extent | – | – | 776 | 61.0 | – | – | 1,037 | 68.4 |  | – | – | 1220 | 49.6 | – | – | 974 | 62.7 |
| Often | – | – | 58 | 4.6 | – | – | 125 | 8.2 |  | – | – | 121 | 4.9 | – | – | 162 | 10.4 |
| Item 7 |  |  |  |  |  |  |  |  |  |  |  |  |  |  |  |  |  |
| Never | 3,585 | 100.0 | 985 | 77.4 | 1,288 | 100.0 | 913 | 60.2 |  | 2,602 | 100.0 | 1373 | 55.8 | 454 | 100.0 | 439 | 28.3 |
| Some extent | – | – | 270 | 21.2 | – | – | 522 | 34.4 |  | – | – | 963 | 39.2 | – | – | 889 | 57.2 |
| Often | – | – | 18 | 1.4 | – | – | 82 | 5.4 |  | – | – | 124 | 5.0 | – | – | 226 | 14.5 |
| Item 8 |  |  |  |  |  |  |  |  |  |  |  |  |  |  |  |  |  |
| Never | 3,585 | 100.0 | 490 | 38.5 | 1,288 | 100.0 | 353 | 23.3 |  | 2,602 | 100.0 | 657 | 26.7 | 454 | 100.0 | 236 | 15.2 |
| Some extent | – | – | 694 | 54.5 | – | – | 954 | 62.9 |  | – | – | 1415 | 57.5 | – | – | 868 | 55.9 |

WF, work-to-family; FW, family-to-work.
