# Supplementary material for: Work–family conflict and self-rated health among Japanese workers: How household income modifies associations
Source: PLoS One. 2017 Feb 16;12(2):e0169903. doi: 10.1371/journal.pone.0169903 (PMC5312934; doi:10.1371/journal.pone.0169903)
Supplement: S2 Table — (DOCX) [file pone.0169903.s002.docx]

**S2 Table.** Odds ratios (OR) for poor self-rated health associated with work–family conflict using limited items, separately by gender

|  | Model 1 | |  | Model 2 | |  | Model 3 | |
| --- | --- | --- | --- | --- | --- | --- | --- | --- |
|  | OR | (95% CI) |  | OR | (95% CI) |  | OR | (95% CI) |
| **Men** |  |  |  |  |  |  |  |  |
| **Work-to-family conflict** |  |  |  |  |  |  |  |  |
| Low | 1.00 |  |  | 1.00 |  |  | 1.00 |  |
| High | 1.82 | (1.59–2.08) |  | 1.84 | (1.59– 2.14) |  | 1.74 | (1.49–2.02) |
| **Family-to-work conflict** |  |  |  |  |  |  |  |  |
| Low | 1.00 |  |  | 1.00 |  |  | 1.00 |  |
| High | 1.89 | (1.65–2.16) |  | 1.88 | (1.63–2.17) |  | 1.75 | (1.51–2.02) |
| **Work Family conflict group** |  |  |  |  |  |  |  |  |
| Low WF and low FW conflicts | 1.00 |  |  | 1.00 |  |  | 1.00 |  |
| Low WF and high FW conflicts | 1.64 | (1.28–2.10) |  | 1.49 | (1.14–1.96) |  | 1.40 | (1.06–1.85) |
| High WF and low FW conflicts | 1.59 | (1.35–1.88) |  | 1.54 | (1.28–1.86) |  | 1.48 | (1.22–1.78) |
| High WF and high FW conflicts | 2.65 | (2.24–3.14) |  | 2.67 | (2.22–3.21) |  | 2.41 | (1.99–2.91) |
| **Women** |  |  |  |  |  |  |  |  |
| **Work to Family conflict** |  |  |  |  |  |  |  |  |
| Low | 1.00 |  |  | 1.00 |  |  | 1.00 |  |
| High | 1.99 | (1.76–2.25) |  | 2.09 | (1.81–2.40) |  | 2.01 | (1.74–2.31) |
| **Family to Work conflict** |  |  |  |  |  |  |  |  |
| Low | 1.00 |  |  | 1.00 |  |  | 1.00 |  |
| High | 1.99 | (1.75–2.25) |  | 1.99 | (1.74–2.28) |  | 1.91 | (1.66–2.19) |
| **Work Family conflict group** |  |  |  |  |  |  |  |  |
| Low WF and low FW conflicts | 1.00 |  |  | 1.00 |  |  | 1.00 |  |
| Low WF and high FW conflicts | 1.91 | (1.60–2.28) |  | 1.79 | (1.46–2.20) |  | 1.70 | (1.38–2.08) |
| High WF and low FW conflicts | 1.93 | (1.60–2.32) |  | 1.88 | (1.53–2.31) |  | 1.79 | (1.45–2.20) |
| High WF and high FW conflicts | 2.94 | (2.51–3.44) |  | 3.05 | (2.56–3.64) |  | 2.90 | (2.42–3.47) |

CI, confidence interval; OR, odds ratio; WF, work-to-family; FW, family-to-work.

Model 1: crude model.

Model 2: adjusted for age, household equivalent income, educational attainment, employment status, and occupation.

Model 3: Model 2 + domestic role, social support, and medical histories of hypertension, diabetes mellitus, and hypercholesterolemia.
